# Chemical Analysis and Evaluation of Antioxidant, Antimicrobial, and Photoprotective Activities of* Schinopsis brasiliensis* Engl. (Anacardiaceae)

**DOI:** 10.1155/2017/1713921

**Published:** 2017-10-15

**Authors:** Sarah Raquel Gomes de Lima-Saraiva, Fernanda Granja da Silva Oliveira, Raimundo Gonçalves de Oliveira Junior, Camila de Souza Araújo, Ana Paula de Oliveira, Alessandra Gomes Marques Pacheco, Larissa Araújo Rolim, Elba Lúcia Cavalcanti de Amorim, Francine Celise Siqueira César, Jackson Roberto Guedes da Silva Almeida

**Affiliations:** ^1^Center for Studies and Research of Medicinal Plants, Federal University of Vale do São Francisco, 56304-205 Petrolina, PE, Brazil; ^2^Federal University of Pernambuco, 50670-901, Recife, PE, Brazil; ^3^University of São Paulo, 14040-900, Ribeirão Preto, SP, Brazil

## Abstract

*Schinopsis brasiliensis* Engl. is a native plant of Caatinga which has high concentrations of compounds capable of absorbing ultraviolet light, suggesting its potential application for the development of sunscreen preparations. After its identification and collection, this vegetable drug was submitted to a physicochemical analysis through the preparation of ethanolic extract. The phytochemical screening and analysis of extracts were carried out by high-performance liquid chromatography (HPLC) evaluation. The antioxidant activity of the extract was evaluated by 2,2-diphenyl-1-picrylhydrazyl (DPPH) method and *β*-carotene bleaching test. Inhibitory hemolytic activity and morphological deformation of erythrocytes induced by H_2_O_2_ were also demonstrated and the antimicrobial activity was analyzed by the minimal inhibitory concentration (MIC) and minimal bactericidal concentration (MBC) method. For the* in vitro* determination of the sun protection factor (SPF), the spectrophotometric method was used. From the analyses carried out with this species, this plant showed significant results for the antioxidant and antimicrobial activities, as well as sunscreen action. Important flavonoids were identified. These data are an important step for the development of new photoprotective cosmetic with Caatinga species, revealing importance and representing another incentive for the preservation of the species involved and analyzed in the study.

## 1. Introduction

The Caatinga is an exclusive Brazilian biome that covers approximately 844 km^2^ of the country, which corresponds to 11% of the national territory. This biome is rich in biodiversity since vegetation has adapted to the semiarid climate conditions and currently more than 1500 endemic species have been registered [1]. Due to its geographical location and its proximity to the Equator, the region receives about 3,000 hours of solar rays incidence per year and its perpendicular incidence allows the region to have marked evapotranspiration indices among other meteorological phenomena. Caatinga also registers high averages of temperature around 26°C [2].

The high solar incidence in this biome interferes directly with the morphology of the vegetation and especially with its chemical composition, since some substances such as flavonoids play a special role in protection against ultraviolet radiation [3].

The sun emits ultraviolet radiation, which is divided according to the wavelengths: UVC (100–290 nm), UVB (290–320 nm), and UVA (320–400 nm). UVC radiation is absorbed by the ozone layer in the stratosphere and represents a low percentage of sunlight on the earth's surface. On the other hand, UVB and UVA radiations are biologically important because they may cause several changes in the skin, such as irregular pigmentation, photoaging, and premalignant and even malignant lesions [4].

The use of sunscreens is an alternative to protect the skin against the incidence of UV radiation. Currently, the development of products with raw materials from natural sources has been reported as a trend due to its good acceptance and the potential activity of natural products [5, 6]; for example, the extracts from the flowers of* Bromelia laciniosa* showed high sun protection factor [7], as well as extracts from the leaves of* Neoglaziovia variegata* [8].

The choice of new photoprotective compounds mainly involves their capacity of ultraviolet light absorption by the chromophores present in the molecules. Generally, structures containing aromatic rings can absorb UV radiation [9]. Thus, there are several natural products with structures containing aromatic rings, such as polyphenols [10].

Several native plant species of Caatinga have high concentrations of these phenolic compounds, such as flavonoids, which are capable of absorbing ultraviolet light and whose absorption spectrum occurs with two peaks, one between 240 and 280 nm and another between 300 and 550 nm. Thus, these plants species may be used for the developments of sunscreens in photoprotective preparations [11]. Therefore,* Schinopsis brasiliensis* Engl. species native to the Caatinga belonging to the family Anacardiaceae, while it was not used popularly as a photoprotective, was selected to study the composition of secondary metabolites potentially applicable for this purpose. Then, the aim of the study was to evaluate the photoprotective effect, antioxidant and antibacterial activity, and chemical analysis of the ethanol extract from the barks of this species.

## 2. Materials and Methods

### 2.1. Plant Material

The plant material was collected in the city of Petrolina in May 2013. Voucher number 2362 of the species was coded and deposited at Herbário Vale do São Francisco (HVASF). The botanical material was identified by comparing the samples collected with a voucher of the species deposited at the HVASF.

After collecting the material, the barks of* Schinopsis brasiliensis* Engl. were dried in circulating air oven at 40°C for 72 h and pulverized in a mill.

### 2.2. Plant Drug Physicochemical Quality Control

Among the characteristics necessary to guarantee the quality of the vegetal material, the recommendations of the Brazilian Pharmacopoeia were followed: foreign material, loss on drying, particle size, total and sulfated ashes, foam index, intumescence index, and hemolytic assay [12].

Additionally, assays were adapted for the determination of pH in aqueous solution, following another study found in the literature [13].

### 2.3. Extraction

The powdered vegetable material was transferred to a flask and subjected to maceration with 95% ethanol, with three extractions at intervals of 72 h, at room temperature, protected from light. The extractive solution was filtered and concentrated under vacuum, and thus the crude ethanolic extract (Sb-EtOH) was obtained [14].

### 2.4. Determination of Total Phenolic Compounds and Tannins

To determine the total phenolic content, the Folin-Ciocalteu method was used and the methodology for precipitation by casein followed by Folin-Ciocalteu was used for tannins. The content of tannins corresponds to the difference between the total and residual phenolic contents [15]. To quantify total phenolic content, aliquots of the sample (1.0 mg/mL) were mixed with 5.0 mL of Folin-Ciocalteu reagent (10%, v/v), 10.0 mL of Na_2_CO_3_ (7.5%, w/v), and 84.0 mL of distilled water. The solution was allowed to stand in the dark for 30 minutes. The absorbance was measured at 760 nm.

To quantify the tannins, aliquots of 10.0 mL of the sample (1.0 mg/mL) were shaken for three hours with 1.0 g of casein. After filtration, the volume was completed to 25.0 mL with distilled water. 2.0 mL aliquots of this filtrate were quantified by the Folin-Ciocalteu method. The correlation equation constructed with tannic acid was *y* = 0.0677*x* + 0.0125 (*R*^2^ = 0.9981). The total phenolic and tannin contents were expressed in milligrams of tannic acid per g of extract (mg EAT/g).

### 2.5. Determination of Flavonoids

The flavonoid content was determined using the colorimetric method based on the formation of the flavonoid-aluminum complex [16]. 2.0 mL aliquots of the sample (1.0 mg/mL) were mixed with 0.6 mL of glacial acetic acid, 10.0 mL of pyridine (20.0%, v/v), 2.5 mL of aluminum chloride (5.0%, w/v), and 10.9 mL of distilled water.

The solution was allowed to stand in the dark for 30 minutes. At 420 nm, the absorbance of the solution was measured. The calibration curve was *y* = 0.0290*x* + 0.0022 (*R*^2^ = 0.9999). The results were expressed in mg of rutin equivalent per g of extract (mg RE/g).

### 2.6. HPLC Analysis

For the qualitative and quantitative determination of secondary metabolites in the extract, a liquid chromatograph (Prominence LC-20AT, Shimadzu®, Japan) with a diode array detector (DAD, SPD-M20A) coupled to an LC Solution ChemStation data-processing station was used.

The mobile phase used was composed of 2 solvents: solvent A (ultrapure water and acetic acid, 0.5%) and solvent B (acetonitrile, 100%) with 1 mL/min flow rate and gradient according to Table 1.

The stationary phase was a TSKgel Super-ODS (Supelco®) column C18 maintained at 30°C. The volume of the samples injected was 20 *μ*L, being monitored at 270 and 340 nm. For quantitative determination of the analytical standards apigenin, catechin, epicatechin, and gallic acid (Sigma-Aldrich®, analytical grade with purity higher than 99%) in the samples, analytical curves were obtained at the concentrations of 50, 100, 125, 150, and 200 *μ*g/mL, and the sample was analyzed at 200 *μ*g/mL in triplicate.

### 2.7. DPPH Assay

The free radical scavenging activity was measured using the 2,2-diphenyl-1-picrylhydrazyl (DPPH) assay [17]. After preparation of the samples (1 mg/mL), concentrations of 1, 3, 9, 27, 81, and 243 *μ*g/mL were then prepared. 2.5 mL of the samples and 1 mL of DPPH (50 *μ*g/mL) were added in glass cuvettes. The solutions were allowed to stand for 30 minutes protected from light, and the samples were measured at 518 nm. Ascorbic acid, BHA, and BHT were used as standards. As a negative control, the DPPH solution was used. The antioxidant activity was calculated from a calibration curve obtained by the percentage of antioxidant activity and expressed as the efficient concentration (EC_50_), that is, the concentration of the sample needed to reduce the absorbance of the 50% negative control.

### 2.8. Inhibition of Autooxidation of *β*-Carotene Assay

The *β*-carotene bleaching method is based on the loss of the yellow color of *β*-carotene due to its reaction with radicals formed by linoleic acid oxidation in an emulsion [18]. *β*-Carotene (2 mg) was dissolved in 10 mL chloroform and linoleic acid (40 mg) and Tween 40 (400 mg) were added to 2 mL of this solution. Chloroform was evaporated under vacuum at 40°C and 100 mL of distilled water was added; then the emulsion was vigorously shaken during two minutes. The emulsion (3.0 mL) was added to a tube containing 0.12 mL of solutions of 1 mg/mL of reference compounds and sample extracts. The reaction was followed by spectrophotometry at *λ* = 470 nm and the test emulsion was incubated in a water bath at 50°C for 120 min, when the absorbance was measured again. The results obtained were compared with the reference antioxidants ascorbic acid, BHA, and BHT and expressed as percentage of antioxidant activity (%AA) using the following formula: % antioxidant activity = [1 − (*A*_0_ − *A*_*t*_)/(*A*_0_^0^ − *A*_*t*_^0^)] × 100, where *A*_0_ is the initial absorbance and *A*_*t*_ is the final absorbance measured for the test sample and *A*_0_^0^ is the initial absorbance and *A*_*t*_^0^ is the final absorbance measured for the negative control (blank). Tests were carried out in triplicate.

### 2.9. Inhibitory Hemolytic Activity and Morphological Deformation of Erythrocytes Induced by H_2_O_2_

Blood was collected and centrifuged at 1500 RPM for 5 minutes; erythrocytes were separated from the plasma and were washed three times using 10 volumes of 20 mM phosphate-buffered saline (PBS), pH 7.4 [19], obtaining a 10% suspension of erythrocytes. To evaluate the antioxidant potential, gallic acid and ethanolic extract samples were used at the concentrations of 5, 10, 15, 20, and 25 *μ*g/mL of total phenol equivalents expressed as gallic acid equivalents. Then, the reaction systems were packed and incubated in an oven at 37°C for 30 minutes. Samples were centrifuged at 2000 RPM for 10 minutes, 500 *μ*L of the supernatant was taken, and 2000 *μ*L of PBS buffer was added. The readings were performed in a spectrophotometer at 410 nm. The percentage of hemolysis was calculated from the ratio between the absorbances of the samples and the negative control.

To evaluate the erythrocyte deformation, a reaction medium containing 50 *μ*L of 10% erythrocytes was used in PBS, 50 *μ*L of standard solution and extract (10 *μ*L) gallic acid equivalents, and 100 *μ*L of 200 mM H_2_O_2_ solution followed by incubation at 37°C for 30 minutes. Samples were centrifuged at 1500 RPM for 10 minutes. The sample was then fixed with 2% glutaraldehyde and centrifuged again. The slide with the material was prepared and then attached to carbon tape in aluminum stub. Scanning electron microscopy (SEM) analysis was performed after metallization with gold powder for 30 seconds.

### 2.10. Evaluation of the Antibacterial Activity

The antimicrobial activity was performed by microdilution method [20]. Solutions of 25 mg/mL of the extract were prepared using 20% (v/v) DMSO and 200 *μ*L of this dilution was transferred to the microplate containing 200 *μ*L of Mueller-Hinton broth. Serial dilutions were then performed, resulting in concentrations of 25, 12.5, 6.25, 3.12, 1.56, 0.78, 0.39, and 0.195 mg/mL. Gentamicin was used as a reference at an initial concentration of 1.6 mg/mL, which was diluted to concentrations of 0.8, 0.4, 0.2, 0.1, 0.05, 0.025, and 0.0125 mg/mL. Control of broth sterility and bacterial growth was also performed.

The inoculum containing 5 × 10^5^ CFU/mL (0.5 McFarland scale) was added to each well and the bacterial strains used in this study came from the National Institute of Quality Control in Health (INCQS/FIOCRUZ, Brazil). The microorganisms used were* Bacillus cereus* (ATCC 11778),* Enterococcus faecalis* (ATCC 19433),* Escherichia coli* (ATCC 25922),* Klebsiella pneumoniae* (ATCC 13883),* Pseudomonas aeruginosa* (Clinical Isolate),* Salmonella enterica* (ATCC 10708),* Serratia marcescens* (ATCC 13880),* Shigella flexneri* (ATCC 12022), and* Staphylococcus aureus* (ATCC 25923). The microplates were incubated under aerobic conditions for 18–24 hours at 37°C, when 10 *μ*L of 2% 2,3,5-triphenyltetrazolium chloride (CTT) was added to each well for detection of the color change of CTT from colorless to red, reflecting active bacterial metabolism. To determine the MBC (minimum bactericidal concentration), 10 *μ*L aliquots were withdrawn from each well containing the extracts and transferred to Petri dishes containing Mueller-Hinton agar. The plates were incubated for 24 hours at 37°C. The appearance of bacterial colonies for a given concentration indicates that it was not able to kill 99.9% or more of bacterial inoculum used. The assays were performed in triplicate.

### 2.11. Determination of the In Vitro Sun Protection Factor (SPF) 

The photoprotective activity of the extract was measured by determination of the maximum absorption wavelength (*λ*max) and* in vitro* sun protection factor (SPF). For this, the samples were diluted in absolute ethanol, obtaining concentrations of 5, 25, 50, and 100 mg/L. Samples were analyzed in triplicate. Subsequently, a spectrophotometer scan was performed at wavelengths between 260 and 400 nm, with intervals of 5 nm. The readings were made using quartz cuvettes, and the ethanol was used as blank [21]. Calculation of SPF was obtained according to the equation proposed by Mansur, and the values of EE × *I* are constant [22].

(1) $$\begin{array}{c} {SPF}_{spectrophotometric}=CF\times \overset{320}{\underset{290}{\sum }}EE\left(\;\lambda \;\right)\times I\left(\;\lambda \;\right)\times Abs\left(\;\lambda \;\right), \end{array}$$

where CF is correction factor (=10); EE(*λ*) is spectrum of the erythematous effect; *I*(*λ*) is solar intensity spectrum; Abs(*λ*) is absorbance.

### 2.12. Statistical Analysis

All determinations were performed in triplicate. Values were expressed as means ± standard deviation and data were considered significantly different at *p* < 0.05. GraphPad Prism® software 5.0 was used, using the one-way ANOVA test, followed by Tukey's test.

## 3. Results and Discussion

The physicochemical quality control of a plant drug is one of the most important parameters before using the plant material for any purpose, as the quality of a natural plant drug should ensure effectiveness and avoid risks to consumers.

In the physicochemical control, the foreign drug materials are classified into three types: (a) parts of the organism from which the drug derives; (b) any organisms, portions, or products of organisms; and (c) impurities of mineral or organic nature, not inherent in the drug. Within this classification, the plant materials were classified as natural, mineral, or organic impurities [12]. From 25 g of the vegetal drug collected by scaling, 0.3 g of foreign material was separated (or 1.2%). This result is within the limits specified by the Brazilian Pharmacopoeia, since the percentage of foreign elements should not exceed 2% m/m.

**Figure 1:**
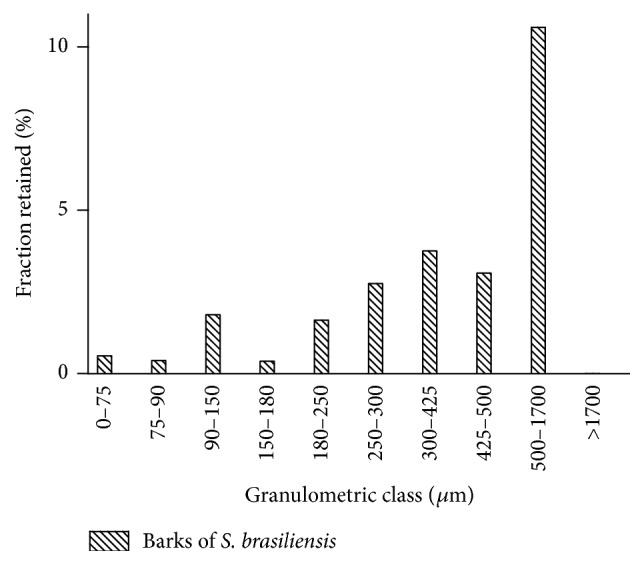
Granulometric distribution histogram of the barks of* Schinopsis brasiliensis*.

The results obtained by the granulometric distribution histogram of the powder of the* Schinopsis brasiliensis* barks demonstrate that the particles of the plant material were found predominantly retained in the 500, 425, and 300 *μ*m mesh, representing, respectively, 42, 12, and 15% of all material (Figure 1).

Following the pharmacopoeia criteria, barks of* Schinopsis brasiliensis* were classified as moderately thick powder [12]. In this context, it should be considered that very fine pulverization is not always viable; there is a risk of the formation of a compact mass, hindering the diffusion of the solvent. Moderately thick powder has advantages for the extraction process, since the adhesion of the particles is difficult, providing better penetration of the solvent, and the use is recommended for most drugs [23].

For the determination of water in plant drugs, the loss on drying method is technically simple and is not applicable when the drug contains volatile substances. The water content is given when a product reaches the thermodynamic equilibrium with air at a given temperature and relative humidity. The amount of water lost by drying was 8.45%. In this context, the water content is considered safe once according to the pharmacopoeia acceptable limits range from 8 to 14% [12].

To quantify the inorganic content of the plant drug, total ash/incineration was performed. Thus, the drug calcined at high temperature has all its organic matter transformed into CO_2_, leaving only mineral compounds in the form of ash [12]. Sulphated ash comprises the nonvolatile residue on incineration in the presence of sulfuric acid. In general, the test aims to determine the content of inorganic impurities contained in organic substances. The averages of the percentage of total ashes were 10.56%. For the content of sulfated ash, the average was 14.50%. The maximum values for total and sulfated ashes vary widely according to the monographs of the drugs described in the Brazilian Pharmacopoeia [12]; for example, in the monograph* Eugeniae folium* the maximum limits described for total ashes and sulfated ashes are 11.0% and 14.0%, respectively. According to the literature, these quality parameters may indicate the presence of nonvolatile inorganic residues such as sand and stone, found naturally since they are samples of plant materials [24].

The erythrocytes are the first point of attack by free radicals due to the polyunsaturated lipids that constitute their cell membrane; these cells are anucleated and thus devoid of repair systems [25]. H_2_O_2_ has a high permeation potential and, with the addition of metal ions, like Fe^2+^, it promotes the formation of the hydroxyl radical (OH^*∙*^), which damages the DNA, RNA, proteins, lipids, and nucleus and mitochondrial membranes [25]. Then, the result of the treatment with the extracts of* Schinopsis brasiliensis* Engl. for the test of inhibitory hemolytic activity and morphological deformation of erythrocytes induced by H_2_O_2_ was positive, since it protected the hemolysis and the deformation of the erythrocytes.

The Sb-EtOH presented a percentage of 43.84% inhibition of erythrocyte hemolysis induced by H_2_O_2_ in the concentration of 15 *μ*g/mL GAE. This result is similar to the value found for the reference substance (gallic acid) at a concentration of 5 *μ*g/mL GAE, as shown in Table 2.

The results in Table 2 show the direct correlation between the total phenol content and the potential for hemolytic inhibition. According to the literature, phenolic compounds at high concentrations have prooxidant potentials and, therefore, the results for high concentrations of the crude extract did not inhibit hemolysis as much, as the increase in total phenol content induced hemolysis rather than preventing it [25–27].

**Figure 2:**
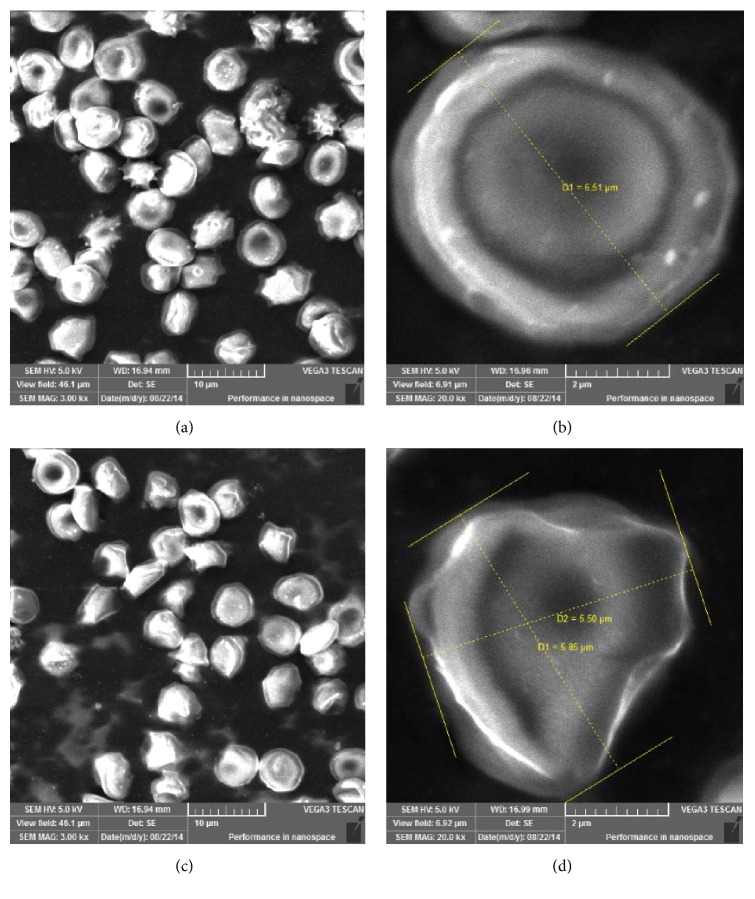
Morphology of erythrocytes after experimental procedure. (a) Sb-EtOH + H_2_O_2_ (10 *µ*m); (b) Sb-EtOH + H_2_O_2_ (2 *µ*m); (c) NaCl 0.9% + H_2_O_2_ (10 *µ*m); (d) NaCl 0.9% + H_2_O_2_ (2 *µ*m). Figures are obtained by scanning electron microscopy.

Regarding the analysis of the erythrocyte morphology, the images obtained by scanning electron microscopy (SEM) show that, in general, the preservation of the erythrocyte morphology by Sb-EtOH did not occur when submitted to attack by the oxidizing agent H_2_O_2_ (Figure 2). The results obtained may be related to phenolic compounds present in the sample [27].

The results of the total phenol content, flavonoids, tannins, and antioxidant activity of crude extract and standards are presented in the Table 3.

Significant contents of total phenolic compounds, flavonoids, and tannins were found in the extract, with 624.6 ± 0.42 (mg EAT/g), 132.4 ± 1.76 (mg RE/g), and 255. 8 ± 2.06 (mg EAT/g), respectively. These results confirm studies previously published with this species [28]. High levels of phenolic compounds were also found in samples of* Schinopsis brasiliensis* [29], in which 455.81 ± 50.41 mg TAE/g were tannins and 11.29 ± 0.94 mg RE/g were flavonoids. The vegetal part used in this study was the barks, while in the literature consulted the leaves were used, which may justify the difference between the results found in the studies.

The Sb-EtOH showed better antioxidant activity then BHT by free radical scavenging method, with a value of IC_50_ of 1.46 ± 0.07. DPPH reacts with an antioxidant compound, which can donate hydrogen and reduce DPPH, promoting change in color from violet to light yellow; therefore, the lower the IC_50_, the better the antioxidant activity [17].

In the *β*-carotene bleaching method, the antioxidant potential of the substance is measured by the ability to sequester the free radical generated during the peroxidation of linoleic acid. The easier its oxidation is, the more it will compete with *β*-carotene in the reaction with radicals, protecting it. The results obtained with the *β*-carotene autooxidation inhibition test indicate that the crude ethanolic extract had 60.81%, lower than BHT and BHA but higher than ascorbic acid, since this method is used to evaluate the antioxidant capacity of nonpolar substances. Antioxidant activity is closely linked to the presence of phenolic compounds, as they have in their structure hydroxyls that can act as electron donor agents. Another structural determinant is the antioxidant capacity of flavonoid attributed to hydroxyls in C4 and C3, which would act to increase the antioxidant potential [30]; these factors contribute to the delocalization of electrons in the aromatic rings, thus allowing the stability of the molecule.

**Figure 3:**
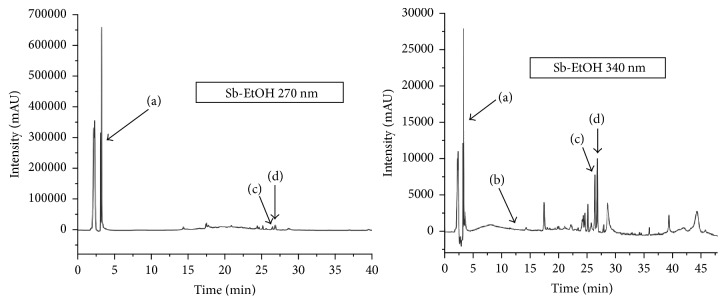
Identification of phenolic compounds in the crude ethanolic extract of the barks of* Schinopsis brasiliensis* using HPLC-DAD (270 and 340 nm). (a) Catechin; (b) epicatechin; (c) apigenin; and (d) gallic acid.

**Figure 4:**
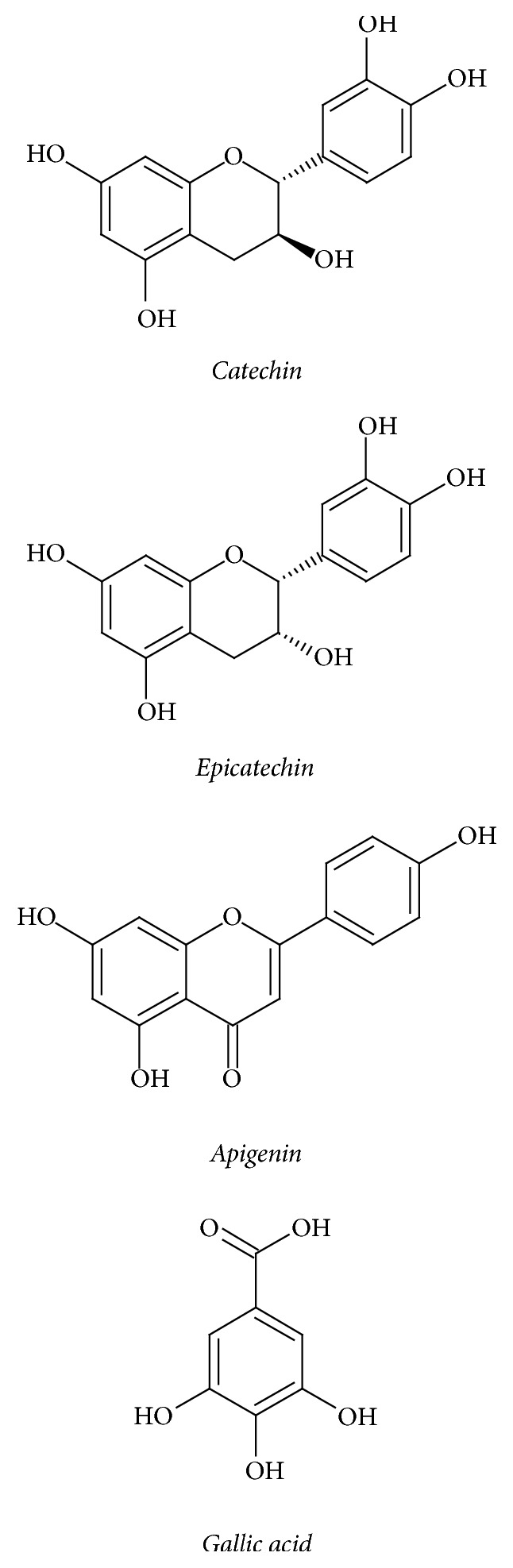
Structures of the substances detected in Sb-EtOH.

Corroborating the results found, it was possible to identify in the Sb-EtOH the four analytical standards used by HPLC-DAD analysis, by means of the similarity between retention times and absorption spectrum in ultraviolet, as well as to quantify them by the compound majority: (a) catechin in 4 min, (b) epicatechin in 3 min, (c) apigenin in 26 min, and (d) gallic acid in 27 min (Figure 3). The chemical structures of these compounds are shown in Figure 4.

The (+)-catechin and (−)-epicatechin are flavanols, which have a hydroxyl group at the 3-position. The properties of the catechins have been widely cited in the literature and present potent sequestering activity of the peroxyl radical about ten times higher than* L*-ascorbate (vitamin C) and *β*-carotene [31].

The apigenin is a flavone that has a carbonyl group at the 4-position and a double bond between the 2,3-positions. Gallic acid has several proven properties, such as anti-inflammatory activity and antimutagenic, antitumor, and antioxidant activity [32, 33].

The antimicrobial activity of the Sb-EtOH was evaluated by determining a small amount of substances necessary to inhibit the growth of microorganisms (MIC) and the minimal bactericidal concentration (MBC). The Sb-EtOH inhibited bacterial growth in all tested microorganisms at concentrations ranging from 12.5 to 0.39 mg/mL (Table 4).

Thus, the extract under study showed antibacterial activity, mainly related to inhibition of growth. In the literature, there are studies that prove this activity, such as the work done with the dry methanolic extract of* Schinopsis brasiliensis* leaf in which it showed good activity against both Gram-positive bacteria and Gram-negative bacteria [25]. The research of new antibiotics in medicinal plants is an important scientific issue due to the increasing incidence of multiple resistances of pathogenic microorganisms to drugs that are currently in clinical use [34].

**Figure 5:**
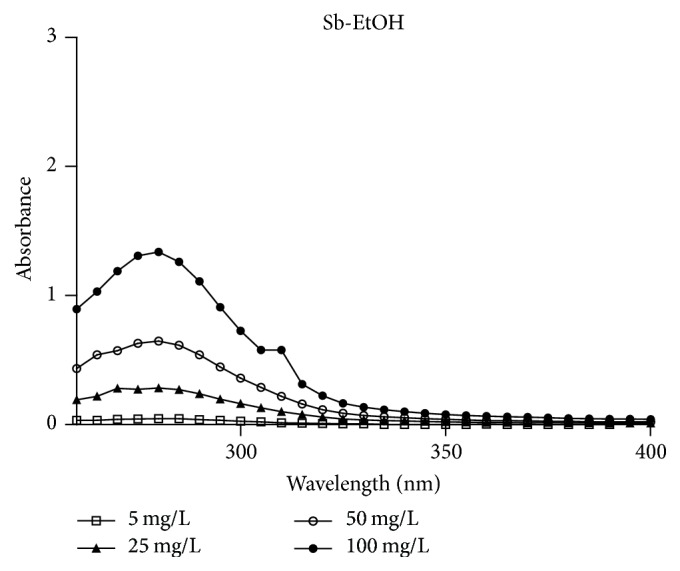
Spectrophotometric absorption profile of* Schinopsis brasiliensis* extract (260–400 nm).

According to the scanning spectrum of Sb-EtOH (Figure 5), it can be observed that the extract analyzed showed a characteristic spectrum of absorption in UVB, since there was absorbance between the wavelengths of 290 and 320 nm. It was found that an increase in absorbance occurred as concentration increases. Thus, Sb-EtOH has a possible photoprotective potential.

Flavones such as apigenin have been described in the literature as being chemical protectors that absorb light at shorter wavelengths than those visible to the human eye, protecting plant cells from the damage caused by photooxidation. This class of flavonoids acts protecting cells against excess UVB (280–320 nm) radiation, as they accumulate in the epidermal layers of leaves and stems and absorb light intensely in this region of UVB [35]. In addition, it has been shown that increasing plant exposure to UVB light results in the increased synthesis of flavones and flavonols.

**Figure 6:**
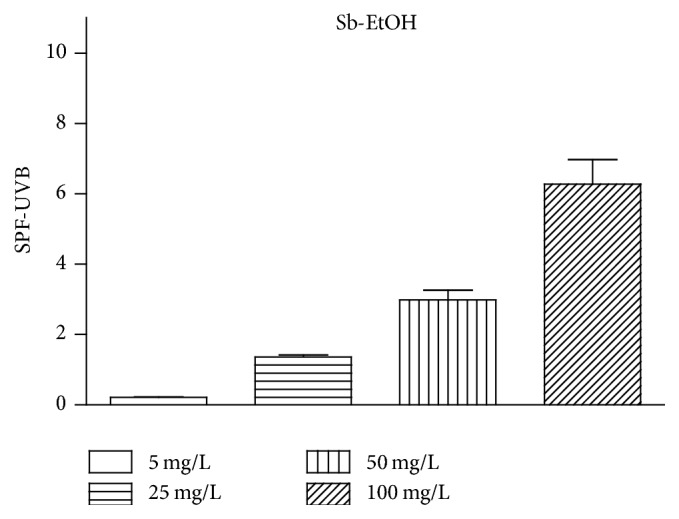
*In vitro* Sun protection factor (SPF) of Sb-EtOH (260–400 nm).

The* in vitro* SPF calculation was determined by the spectrophotometric method [18] using the UVB region. The SPF values of Sb-EtOH [5, 25, 50, and 100 mg/L] are, respectively, 0.21 ± 0.01, 1.35 ± 0.05, 2.98 ± 0.27, and 6.27 ± 0.69. The results are shown in Figure 6.

According to Resolution RDC 30/12 [36] published by ANVISA (National Health Surveillance Agency), the minimum value of sun protection factor for products used by the Brazilian population is 6 (SPF 6). Thus, the Sb-EtOH fulfills this prerequisite of minimum values for the values of photoprotection. This result can be attributed to the presence of flavonoids in the sample.

Although the test was performed* in vitro*, this method correlates well with the* in vivo* tests because it refers to the absorbance of the substance with the erythematogenic effect of radiation and light intensity at specific wavelengths between 290 and 320 nm (UVB region).

## 4. Conclusions

The physicochemical analysis showed that the vegetal drug of* S. brasiliensis* is within the limits specified in the Brazilian Pharmacopoeia. The chemical analysis showed that the ethanolic extract studied contains significant amount of phenolic compounds, specifically flavonoids and tannins. Important flavonoids were identified by HPLC-DAD analysis and this study indicated that this plant extract has potent antioxidant and antimicrobial activities, as well as sunscreen action, important data for the future development of a new photoprotective formulation.

## Figures and Tables

**Table 1:** Mobile phase gradient used for determination of secondary metabolites.

Time (min)	Solvent A (%)	Solvent B (%)
0.00	100	0
40.00	20	80
50.00	100	0

**Table 2:** Percentage of maximum inhibition of erythrocyte hemolysis induced by H_2_O_2_.

Sample	% maximum inhibition ± MSE
Sb-EtOH	43.84 ± 0.02
Gallic acid	50.68 ± 5.48

MSE: Mean Standard Error.

**Table 3:** The content of total phenols (FT), flavonoid (F), tannins (T), and antioxidant activity were determined by DPPH (EC_50_) and *β*-carotene (% AA).

Sample	Results			Antioxidant activity	
	TP (mgGAE/g)	F (mgRE/g)	T (mgTAE/g)	DPPH (EC_50_)	*β*-Carotene (% AA)
Sb-EtOH	624.6 ± 0.42	132.4 ± 1.76	255.8 ± 2.06	1.46 ± 0.07	60.81 ± 0.67
Ascorbic acid	—	—	—	0.64 ± 0.22	7.50 ± 2.12
BHA	—	—	—	1.22 ± 0.43	97.49 ± 0.76
BHT	—	—	—	8.16 ± 7.06	99.10 ± 1.14

GAE: gallic acid equivalent; RE: rutin equivalent; TAE: tannic acid equivalent. The IC_50_ values were obtained by linear regression with 95% confidence interval. IC_50_ is defined by the concentration sufficient to obtain 50% of the maximum estimated effect at 100%. Values are expressed as mean ± SD (*n* = 3).

**Table 4:** Antimicrobial activity of the extract of *Schinopsis brasiliensis.*

Bacteria	Sb-EtOH (mg/mL)	
	MIC	MBC
*Bacillus cereus*	12.5	—
*Escherichia coli*	12.5	12.5
*Enterococcus faecalis*	12.5	12.5
*Klebsiella pneumoniae*	12.5	12.5
*Pseudomonas aeruginosa*	12.5	—
*Serratia marcescens*	6.25	12.5
*Shigella flexneri*	3.12	12.5
*Staphylococcus aureus*	3.12	12.5
*Salmonella enterica*	0.39	12.5
